# Development of 7TM receptor-ligand complex models using ligand-biased, semi-empirical helix-bundle repacking in torsion space: application to the agonist interaction of the human dopamine D_2_ receptor

**DOI:** 10.1007/s10822-013-9640-z

**Published:** 2013-04-04

**Authors:** Marcus Malo, Ronnie Persson, Peder Svensson, Kristina Luthman, Lars Brive

**Affiliations:** 1 Department of Chemistry and Molecular Biology, University of Gothenburg, SE-412 96 Göteborg, Sweden; 2 NeuroSearch Sweden AB, Arvid Wallgrens Backe 20, SE-413 46 Göteborg, Sweden; 3 Department of Medical Biochemistry and Cell Biology, University of Gothenburg, Box 440, SE-405 30 Göteborg, Sweden; 4 Cygnal Bioscience, Björnvägen 15, SE-435 43 Pixbo, Sweden; 5 Present Address: Astra Zeneca R&D Mölndal, SE-431 83 Mölndal, Sweden

**Keywords:** GPCR, 7TM, Membrane protein, Dopamine D_2_ receptor, Structure prediction, Receptor modeling, Agonist, Ligand binding

## Abstract

Prediction of 3D structures of membrane proteins, and of G-protein coupled receptors (GPCRs) in particular, is motivated by their importance in biological systems and the difficulties associated with experimental structure determination. In the present study, a novel method for the prediction of 3D structures of the membrane-embedded region of helical membrane proteins is presented. A large pool of candidate models are produced by repacking of the helices of a homology model using Monte Carlo sampling in torsion space, followed by ranking based on their geometric and ligand-binding properties. The trajectory is directed by weak initial restraints to orient helices towards the original model to improve computation efficiency, and by a ligand to guide the receptor towards a chosen conformational state. The method was validated by construction of the β_1_ adrenergic receptor model in complex with (*S*)-cyanopindolol using bovine rhodopsin as template. In addition, models of the dopamine D_2_ receptor were produced with the selective and rigid agonist (*R*)-*N*-propylapomorphine ((*R*)-NPA) present. A second quality assessment was implemented by evaluating the results from docking of a library of 29 ligands with known activity, which further discriminated between receptor models. Agonist binding and recognition by the dopamine D_2_ receptor is interpreted using the 3D structure model resulting from the approach. This method has a potential for modeling of all types of helical transmembrane proteins for which a structural template with sequence homology sufficient for homology modeling is not available or is in an incorrect conformational state, but for which sufficient empirical information is accessible.

**Electronic supplementary material:**

The online version of this article (doi:10.1007/s10822-013-9640-z) contains supplementary material, which is available to authorized users.

## Introduction

The family of monoaminergic G-protein coupled receptors (GPCRs) is well-studied due to their relevance as drug targets. For a complete understanding of the detailed mechanism for ligand interaction with these receptors, access to accurate and reliable 3D structures is needed. GPCRs are believed to exist in active signaling states stabilized by agonists, and in inactive states stabilized by inverse agonists [1, 2]. The high affinity state has been shown to be associated with the functional state of the receptor which activates the G-protein and induces downstream signaling [1, 3]. Solved 3D structures of GPCRs include several structures of rhodopsin with (e.g. Refs. [4, 5]) and without (e.g. Refs. [6, 7]) covalently bound *trans*-retinal, the inactive states of the turkey β_1_- [8] and human β_2_- [9, 10] adrenergic receptors (β_1_AR and β_2_AR), the human A_2A_ adenosine receptor with a bound inverse agonist [11], and the CXCR chemokine receptor [12] and dopamine D_3_ receptor with an antagonist [13]. For a recent review on all GPCRs of known structures, see Reference [14]. Only recently have structures of active- or near-active-state GPCRs in the presence of agonists been determined, achieved using an A_2A_ adenosine receptor—T4L chimera bound to UK432097 [15], thermostabilized A_2A_ adenosine receptors bound to adenosine and NECA [16], or by using fragments of antibodies to stabilize the agonist-bound state of the β_2_AR [17, 18]. These structures confirmed previous hypotheses [19–22] that the agonist-bound active-state binding site is contracted by 1–2 Å relative to that bound to structurally related inverse agonists. The major conformational changes, however, occur on the intracellular side where transmembrane helices 5 and 6 (TM5 and 6) are extended and move outwards to allow binding of the G-protein.

Although several examples of family A GPCR structures have recently appeared in the literature, their experimental structure determination is time-consuming and difficult, which makes access to modeling techniques highly desirable. A protein structure in a particular conformational state can be used to predict the structure of another protein of sufficient sequence homology in that same state using homology modeling. Three-dimensional models that can identify antagonists in virtual ligand screening (VLS) experiments have been constructed by inclusion of QSAR data [23], and new micromolar antagonists have been discovered based on VLS using a structure model where binding pocket side chains were optimized with a ligand present [24] or by repacking of the transmembrane part [25]. Tang et al. [26] reported that manually refined homology models may be on par or even better than crystal structures for VLS. For the majority of GPCRs, however, the sequence identity within the family is generally low [27], and only few structures of GPCRs in an active, agonist-bound state have been reported. In addition, the structural diversity of solved GPCR structures, mainly in loop regions and at the intracellular side, shows that homology modeling of remote homologs will be challenging. An additional complication is that GPCRs bind ligands through multiple conformational states. Therefore, the inactive-state crystal structure of the β_2_ adrenergic receptor (β_2_AR) was not able to represent the interactions with agonists [19], and the identification of agonists by VLS using homology models based on inactive-state structures was only possible after careful structural refinement (see e.g. refs. [21, 28, 29]).

Methods for ab initio prediction of receptor structure aim to circumvent the problem of lack of closely related template structures. Transmembrane helices are constructed from the amino acid sequence, followed by their assembly into a helix bundle guided by data from known structures. Several approaches have been described: Yarov-Yarovoy et al. [30] adapted the ROSETTA structure prediction method for membrane proteins, and applied it to 12 diverse membrane proteins. Goddard and coworkers developed MembStruk and applied it to the prostaglandin D [31], β_2_AR [32] and dopamine D_2_ [33] (D_2_R) receptors. Shacham et al. [34] developed the PREDICT approach to model the D_2_R, the neurokinin NK1 and neuropeptide Y1 receptors. Other studies describe methods where homology models are modified in a systematic way to overcome the lack of appropriate templates. For example, Evers and Klebe [23] reported an iterative homology model building method including ligand restraints which was used to produce an NK1 receptor model that allowed the identification of a compound that inhibited substance P binding. Michino et al. [35] recently reported a method that reproduced the rhodopsin and β_2_AR/carazolol structures to approximately 2–2.5 Å C_α_ RMSD by restrained molecular dynamics simulation of the helical regions. We have previously modeled dopamine D_2_ [36] and D_1_ [37] receptors using homology modeling with an agonist present in the binding site during the procedure. The model RMSD for C_α_ in the TM region relative to the template structure (β_2_AR, pdb code 2rh1 [19]) was 1.9 Å and 1.5 Å for the D_1_ and D_2_ receptor models, respectively.

We present here a new method to generate all-atom models of the membrane-spanning part of TM proteins that repacks secondary structure elements of a homology model guided by a ligand and a limited set of experimental and evolutionary restraints. The rationale is to allow models to deviate more from the template structure than homology modeling does, while including experimental restraints based on other experimental data in order to make the conformational search efficient. An initial homology model is subjected to random helix displacements and Monte Carlo geometry optimization to generate a large number of receptor conformations from which the most probable candidates are selected by means of a scoring scheme. The method contains several elements of *ab inito* protein structure modeling, but also uses restraints of experimental origin, and is therefore referred to as a semi-empirical approach. An agonist was present in the binding site during the modeling to focus sampling towards the agonist-bound conformation. The main goal of the current study was to analyze ligand binding to the D_2_R binding site, and therefore selected models were further evaluated by docking of 29 compounds with known pharmacological profiles towards the D_2_R.

## Results and discussion

### Helix docking method

In the present study, the receptor structure prediction was based on the docking of seven individual helices (TM1–7) that were initially rigid but gradually made more flexible as structures became more refined. A homology model of the transmembrane helices was used as the starting model, numerous copies were created and their helix coordinates were perturbed according to a defined stochastic scheme to expand the covered conformational space, and brought back to a compact shape by a Monte Carlo geometry optimization (see below for details). A ligand was present during the helix packing optimization to direct the bundle towards a biologically relevant structural state, for example agonist or inverse agonist-induced states.

Intra- and extracellular loops were removed to make the conformational sampling more efficient, and also because loops are notoriously difficult to predict as they vary both in length and sequence [38]. The loops can be added back to the helical bundle once the preferred solution (or solutions) has been found. Although the second extracellular loop is crucial for ligand discrimination in some receptors, e.g. D_2_R [39–41], the present study focuses on the TM region which contributes the majority of ligand contacts. In addition, incorrect modeling of loops may have an adverse effect on the results. Removal of loops in the β_2_AR structure did not prevent the correct docking of carazolol [27], and it has been shown that ligand docking has in fact given equal or better results with the loops excluded [42].

The procedure is described in general terms below, followed by a description of the scoring method, the validation of the method by building of the β_1_AR, and finally an application of the method to the D_2_R. A multiple sequence alignment of relevant sequences was performed, followed by manual editing guided by the 3D structures. Typically, the modeling template (or templates) would be chosen based on multiple factors, including the quality of the pairwise alignments, the conformational state and quality of the structures, and the structure of the ligand. Structures of monoaminergic receptors are available for modeling the D_2_R. However, we wanted to evaluate the prediction method based on a more remote homolog, and therefore chose bovine rhodopsin as template. The starting structure was created from the helical regions of the template structure using the modeling software ICM (ICM v 3.4, Molsoft LLC, CA), where the exact sequence positions of helix termini were assessed manually to take sequence alignments and 3D-structure into account.

**Fig. 1:**
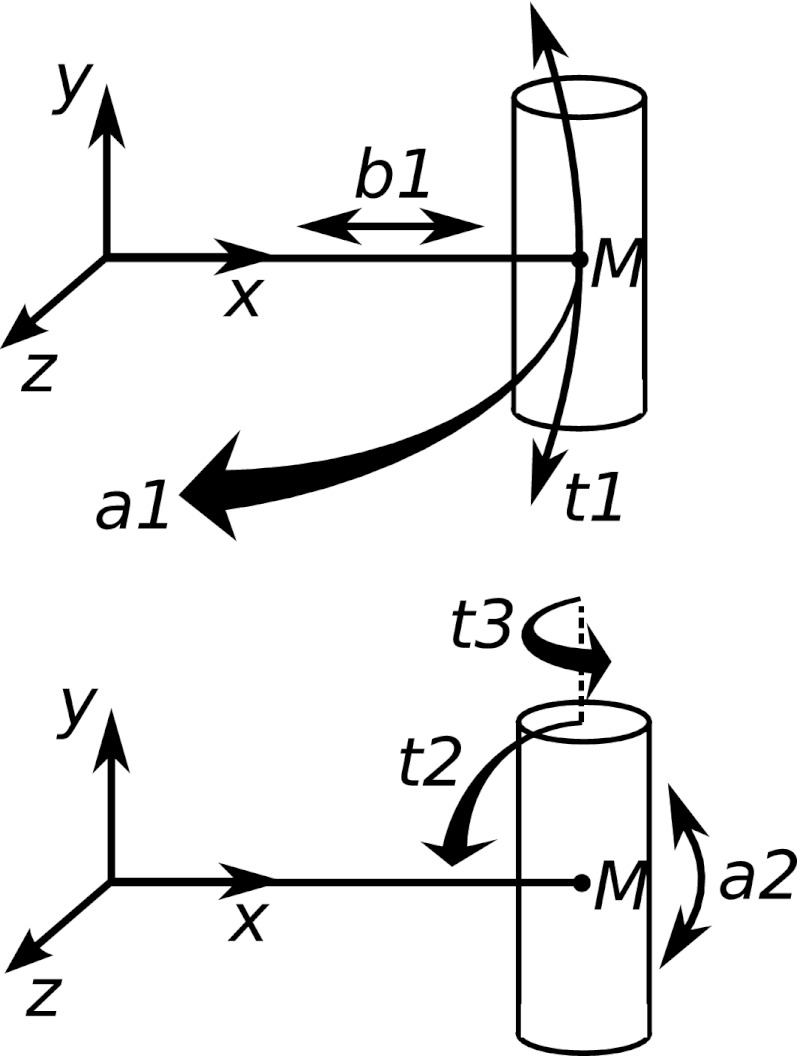
The overall position and orientation of a helix is determined by six variables in the internal coordinate space representation, which simplifies molecular transformations (e.g. controlled randomization of helix positions) and makes geometry optimizations more efficient [62]. Labels indicate the N-terminus (N), the center of mass of the helix (M), the coordinate system axes (*x*, *y*, *z*) and virtual variables (*a1*, *b1*, *t1*, *a2*, *t2*, *t3*)

In order to decrease the dependence on the template model and cover a larger conformational space, many copies of the helix bundles were made and each was expanded and randomly displaced, i.e. each helix of a model was moved by a random distance (0–5 Å) away from the bundle center in the membrane plane, tilted with respect to its center (0 ± 20 °) and rotated around the helical axis (0 ± 30 °). These values were derived by observing the effects of different settings, and were found to allow a proper sampling of the conformational space while avoiding the generation of unrealistic starting structures. The molecular system was defined in internal coordinate space which conveniently allows each of these geometrical properties of a helix to be controlled by a single variable (Fig. 1).

A ligand was placed at least 5 Å away from the helix bundle and its positional and internal torsion variables were randomized to ensure that the model was not biased towards the starting geometry and position of the ligand. Rigidity of the ligand reduces the risk that less realistic complex models are generated due to incorrect ligand geometry. A minimal number of loose distance restraints were used to orient the ligand relative to amino acids that are known to be important for ligand binding in the initial optimization phases, as described in detail for each target below. The purpose was to exclude docking conformations that disagree with available data and generally accepted concepts of receptor-ligand interactions.

**Fig. 2:**
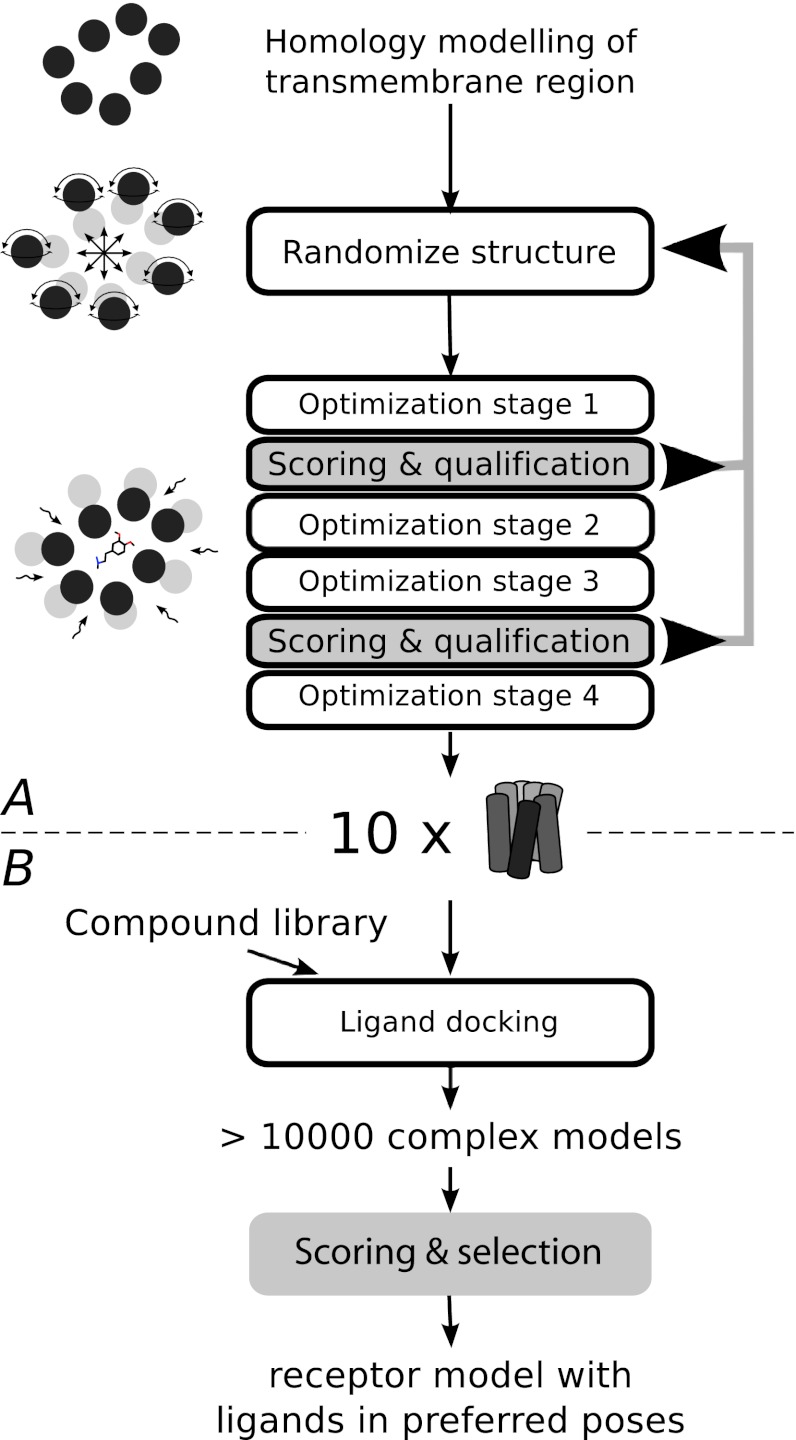
Overview of the generation of ligand-receptor models from a homology model. The iterative process in A is increasingly CPU demanding, allowing gradually more degrees of freedom, stronger van der Waals interactions, and a decreased number of restraints. Therefore, the results are scored at two stages so non-productive solutions can be dismissed at early stages. In **B**, a library of compounds is docked to the ten receptor models from **A** using the standard ICM protocol. The model(s) that best matches binding data is selected for analysis

Monte Carlo geometry optimization brought the bundle back to a compact shape in four main stages. The first stage was a rough optimization of rigid helices and rigid ligand, followed by three optimization stages with increasing level of detail and demand for computation resources (Fig. 2). The main changes of the procedure during the process were the following: (1) The number of free variables was increased stepwise. Sets of torsion angles were gradually made flexible such that the final optimization was performed over all torsion variables, including those of the backbone. (2) Regions of high sequence conservation were expected to be more structurally conserved, and the optimization was therefore biased towards the starting structure by the use of distance restraints to the homology model (tethers) for strictly conserved residues with a target value of 0 Å. The strength of the tethers can be tuned such that computational resources are not spent on sparsely packed solutions (too weak restraints) while avoiding the regeneration of the starting structure (too strong restraints). Tethers were gradually softened and completely turned off during the longest, final optimization stage. (3) van der Waals interactions were soft in the initial phase to decrease steric repulsion energies of the coarse models and increased gradually to standard Lennard-Jones 6–12 potentials.

We noticed that models that were geometrically unacceptable at an early stage rarely resulted in satisfactory models at the final stage. A scoring method (see the “Methods/experimental” section) was therefore developed that evaluated the geometry of each model after stage 1 and 3. To probe how the scores varied as a function of simulation time, intermediate geometries of a limited number of structure models were evaluated during the optimization stages (Supplementary Fig. 1). It was concluded that the majority of the final high-scoring models were recovered even if 50 % of the models were discarded earlier during the optimization. By choosing proper score thresholds, models are eliminated after the first and third stage which dramatically improves performance since the later stages are the most computationally intensive. The homology model geometry perturbation and first optimization stage are fast, and the first threshold is therefore set at a high score value to produce a large pool of conformations to be evaluated. When a selected number of final models are available at the second threshold, the models are submitted to the final fourth stage optimization.

The total number of models that are created depends on the selected thresholds and on the selected number of final models. For the complexes in this study, hundreds of models were typically produced after rigid docking of helices with ligand present (stage 1, Fig. 2), dozens at the second and third stage, and around 10 models selected for the final optimization.

### Generation of β_1_AR models from rhodopsin

**Fig. 3:**
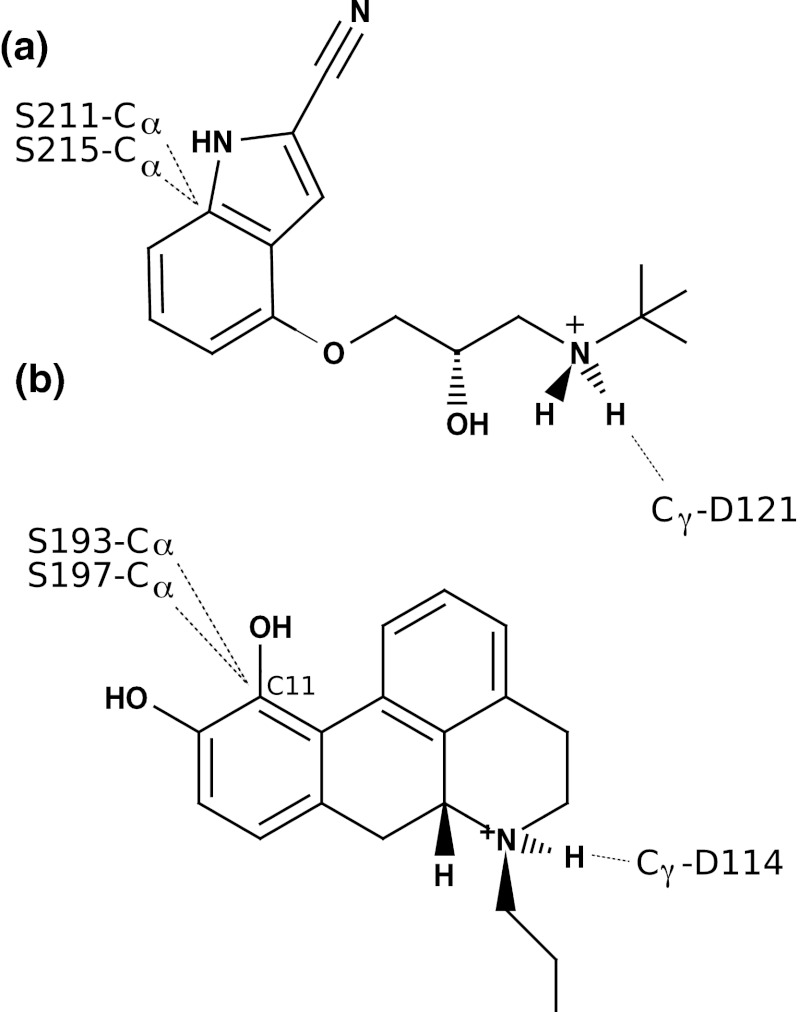
Distance restraints between the ligand and receptor place the ligand near the binding pocket and promote the correct length-wise orientation during the geometry optimization. a) Three loose distance restraints roughly orient (*S*)-cyanopindolol in the β_1_AR model binding pocket. Atoms were chosen to minimize bias with regards to the exact orientation. Hence, C_α_ atoms of S211/5.42 and S215/5.46 allow either or both side chain or backbone oxygens to form hydrogen bonds. b) Three distance restraints roughly orient (*R*)-NPA in the D_2_ receptor, in analogy to those in panel a. Two restraints include α-carbons of serine residues in TM5 to carbon C11 in the ligand to allow any of the main-chain and side chain oxygen atoms to hydrogen bond to either or both catechol oxygens

The turkey β_1_AR receptor structure [8] in complex with (*S*)-cyanopindolol was used to assess the structure prediction method. A homology model was built based on the 2.2 Å resolution crystal structure of bovine rhodopsin bound to the inverse agonist *cis*-retinal (pdb ID 1u19 [5]), and the inverse agonist (*S*)-cyanopindolol in its protonated form was generated from 2D coordinates and added to the receptor model, using ICM. Two (*S*)-cyanopindolol atoms were restrained to receptor residues to ensure the correct length-wise orientation while avoiding the bias for the exact local geometry (Fig. 3). The aim was to apply the method to the D_2_R, and therefore the restraints were selected to mimic those of the D_2_R case to ensure that the results were comparable. Thus, the source of the restraints was based on D_2_R experimental data, as described below.

A total number of 364 models were generated: 270 (stage 1), 42 (stages 2 and 3), and 10 (stage 4). The RMSDs for C_α_ atoms of the models compared to the known β_1_AR receptor structure (chain A of PDB ID 2vt4, Warne et al. [8]) was between 2 and 6 Å for 92 % of the solutions (Supplementary Fig. 2), and from 2.4 to 5.5 Å for the ten stage 4 solutions (Supplementary Table 1). The corresponding RMSD of the homology model was 2.9 Å. Three out of ten models had RMSD values lower (better) than that of the homology model with respect to all C_α_ atoms, and three models had lower RMSDs for the binding site heavy atoms.

**Fig. 4:**
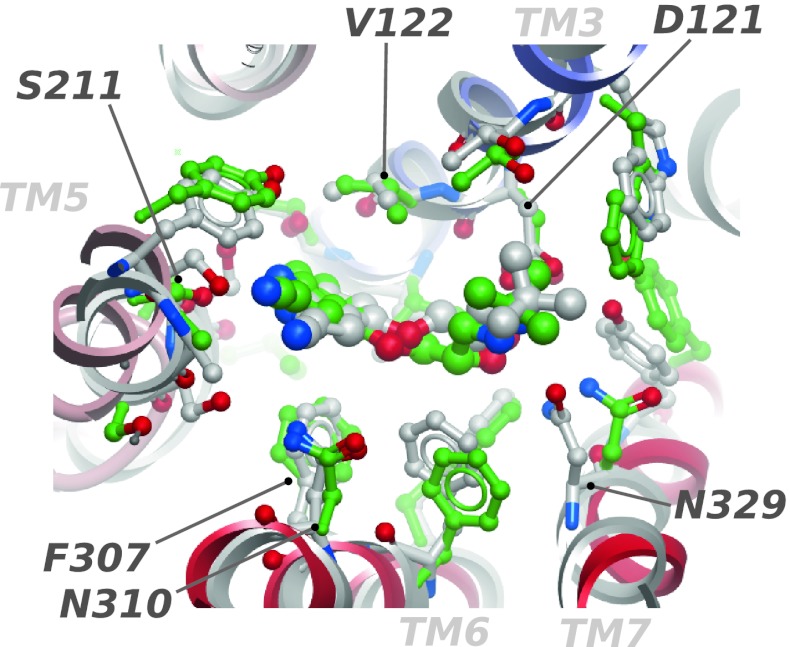
Structural superposition of the binding site residues of the β_1_AR X-ray structure (*white ribbon* and carbon atoms) and the model that best reproduces the bound ligand conformation (*colored ribbon and green carbons*) viewed from the extracellular side. This region of the predicted model matches well that of the crystal structure, and all receptor—ligand hydrogen bonds are reproduced except that between the carbonyl oxygen of N329/7.39 and the basic nitrogen

In order to evaluate the docking of compounds to the models, the ligand was removed and (*S*)-cyanopindolol was re-docked to each stage 4 receptor model in triplicate (Supplementary Table 1). The lowest RMSD value for the ligand (0.5 Å, determined after superposition of receptor binding pocket residues as described in the “Methods/experimental” section) was observed for the model that ranked two in total score and five in binding site score (Supplementary Table 1, see the “Methods/experimental” section for score definitions). The heavy atoms of the receptor binding site, as defined by residues within 5 Å from the ligand in the crystal structure, had an RMSD of 1.6 Å for this model (Fig. 4). The docked ligand reproduces the receptor interactions well including all hydrogen bonds except that between the N329/7.39 oxygen and the basic nitrogen, and a non-optimal interaction between the protonated ligand nitrogen and D121/3.32 (residues are referred to by their position in the sequence followed by the numbering according to Ballesteros-Weinstein [43]). However, the N329/7.39 interaction with the β-hydroxyl group of the ligand is in place. The high total C_α_ RMSD of 5.5 Å for this model is due to incorrect TM1 and TM4 positions, and the lack of a helical kink of TM1 (at residue L50) which is unique to the A and D chains of the crystallographic structure of β_1_AR. Helices TM1 and TM4 are expected to be more arbitrarily positioned as they have lower sequence conservation to the template which leads to fewer restraints. In fact, TM1 shows more structural variability when compared to the other TM helices in determined GPCR structures [14]. In addition, the incorrect positions of TM1 and TM4 will not be penalized by the ligand score and has no direct effect on the binding site geometry as they are not in direct contact with the ligand.

Four additional models reproduce the correct ligand binding conformation to 1.4–1.7 Å RMSD, showing that the method generates several models that are relevant for interpretation of ligand recognition. This is a clear improvement over the RMSD results of the corresponding starting homology model, which were 3.0, 6.5 and 7.1 Å (Supplementary Table 1). Additional information on ligand binding, e.g. mutation data pointing to critical interactions, should be used to select the preferred solution among the top ranked candidates. The low RMSDs for the receptor and ligand, and good representation of the essential receptor-ligand interactions for β_1_AR demonstrate the ability of the structure prediction method to generate relevant structure models of monoaminergic receptors using bovine rhodopsin as template.

### Generation of the human dopamine D_2_ receptor model

The helix docking protocol was also applied to the human D_2_R. The starting structure was a homology model for the helical regions based on the same crystal structure of bovine rhodopsin bound to the inverse agonist *cis*-retinal (PDB id 1u19 [5]) as was used for β_1_AR. The sequence identity for the selected region is 25 % (Supplementary Fig. 3). Tethers were set to the α-carbons of the homology model for residues that are conserved between the model and the template. The average number of restraints per helix is 7, or broken down per helix: 3 (TM1), 10 (TM2), 6 (TM3), 5 (TM4), 10 (TM5), 11 (TM6) and 4 (TM7).

In the helix docking procedure, the D_2_R-selective full agonist (*R*)-*N*-propylapomorphine ((*R*)-NPA) was included to bias the simulation towards the agonist-bound state. Three loose distance restraints were applied to roughly orient the ligand in the binding pocket with the protonated amine near D114/3.32 and the catechol ring near S192/5.42 and S197/5.46, based on experimental data (reviewed in [44]) (Fig. 3). Although either one or both ligand catechol oxygen atoms participate in hydrogen bonding to S193/5.42 and S197/5.46 side chain hydroxyl groups in TM5 [45] (or main chain carbonyl oxygen atoms), the restraints were set to carbon atom C11 (Fig. 3) in order to reduce the structural bias and to improve conformational sampling.

Experimentally determined ligand-receptor interactions were included in the ligand scoring scheme: Electrostatic and hydrogen bonding energies were evaluated for the salt bridge between the protonated amine and the aspartic acid residues in TM3, and for interactions between serine residues in TM5 and heteroatoms of the ligand (see the “Methods/experimental” section for details, and Ref. [44] for a review of the interactions). Complex models that did not contain a hydrogen bond-stabilized salt bridge were excluded. It has also been proposed that aromatic interactions between F390/6.52 and the catechol moiety are important for agonist binding (see e.g. Refs. [46, 47] and references therein). Since aromatic edge-to-face π-interaction energies (reviewed by Waters [48]) are difficult to evaluate using molecular mechanics methods, we verified instead that aromatic groups were in contact by (1) calculating the van der Waals intermolecular interaction energy between aromatic atoms of F390/6.52 and the ligand and (2) discarding solutions with energies higher than −0.3 kcal/mol. With the chosen threshold value, the results correlate well with the results from manual inspection of the complexes.

A total of 472 models were generated at stage 1, whereof 38 passed the first selection filter and nine the second filter. Despite the use of tethers during the initial steps, the C_α_ RMSD was 3–12 Å demonstrating the wider sampling of conformational space. Since the overall bundle geometries were adequate for the nine final models, a more detailed criterion for selection was needed that focused on the properties of the binding pocket. Binding data is available for a number of D_2_R ligands (Supplementary Table 2), which allows the docking and scoring of compounds to define a second model selection criterion, as described below. It is clear that GPCRs are dynamic and probably bind structurally diverse ligands by adopting different conformations [49]. We previously studied agonist binding to D_2_R [36, 40], and in the present study we therefore focused on full agonists and inactive compounds.

### Selection of dopamine D2 receptor ligands

In virtual ligand screening, docking of a compound library to a receptor structure model is typically carried out to rank compounds for their propensity of binding to the receptor. We assumed that an opposite approach is also valid: By docking of a library of compounds containing both binding and non-binding compounds and measuring their geometric fit to several receptor models, the models can be ranked for their ligand binding predictive ability. We therefore selected compounds from the literature with known affinity and intrinsic activity for the D_2_R.

**Fig. 5:**
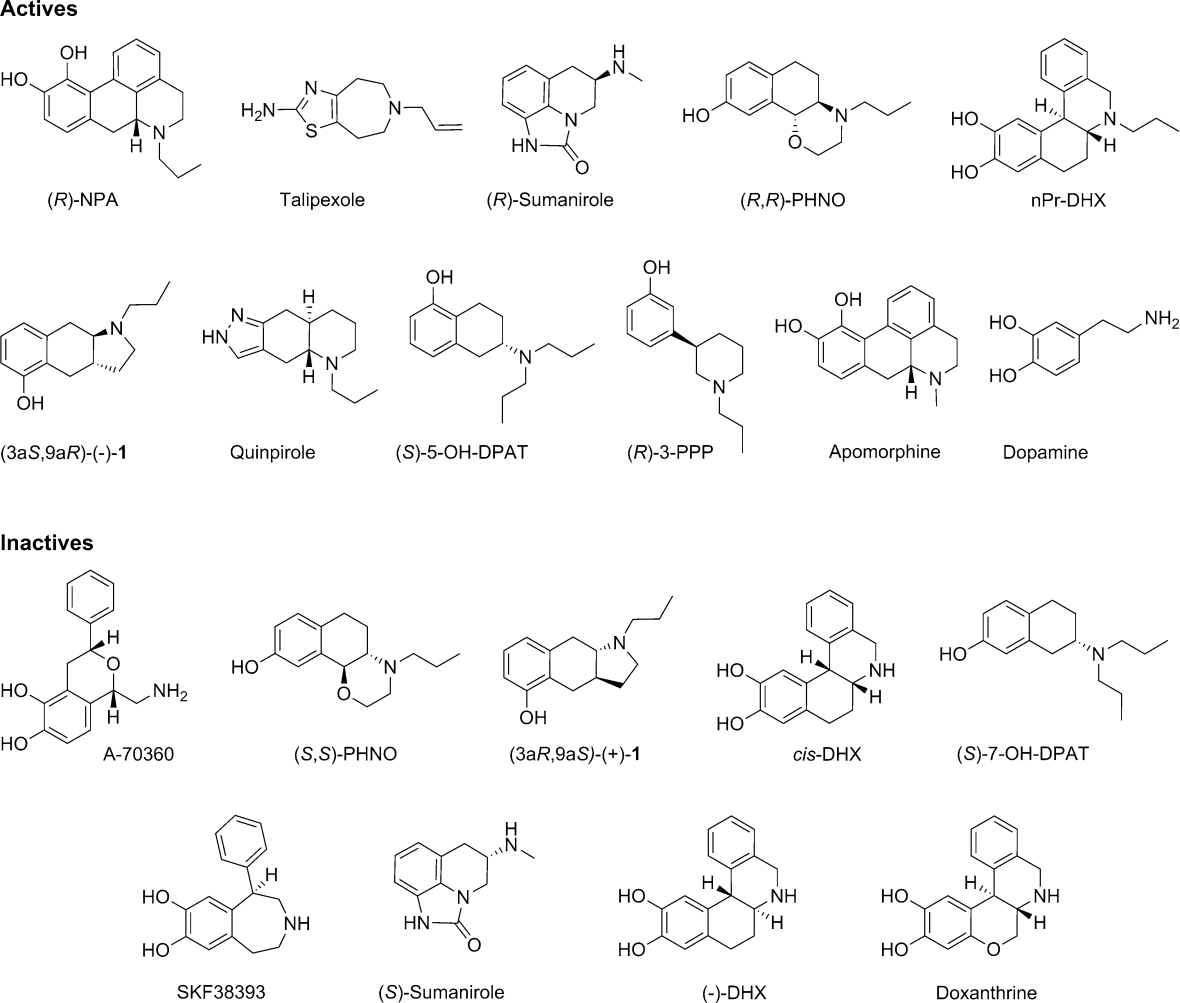
Structures of full D_2_ agonists and inactive compounds used for docking to the final, selected D_2_R model. References and structures used in the docking to all D_2_ receptor models are listed in Supplementary Table 2

The series of compounds used in this study belong to different structural classes comprising the basic dopamine skeleton in their structure, such as aminotetralins, phenethylamines, apomorphines, and benzoquinolines. The ligands were selected on criteria related to their intrinsic activity, selectivity, conformational flexibility and stereochemistry (Fig. 5 and Supplementary Table 2). Sufficiently rigid and selective full agonists at the D_2_R have been chosen in the modeling together with structurally related inactive compounds found in the literature. Inactive analogs are represented by compounds which do not show any or only weak effects, i.e. inverse agonists, antagonists or low intrinsic partial agonists.

The final set contains 29 compounds (Fig. 5 and Supplementary Table 2). A full account for most compounds in the set is described elsewhere [40]. Basic nitrogen atoms were protonated before docking. The stereochemistry of protonated tertiary amine is important in the protein complex model and thus both “*N*-enantiomers” were included, resulting in a total of 43 compounds in the docking set.

### Ranking of receptor models by D_2_ ligand docking performance

The set of compounds was docked to all nine generated receptor models using torsion space Monte Carlo optimization to potential (grid) maps representing van der Waals, electrostatic, hydrogen bonding and hydrophobic interactions that substitute for the receptor, as implemented as the standard protocol in ICM (version 3.4). Several docking solutions were stored for each ligand (typically 30–40) by the protocol. The lowest-energy conformation did not always make the key interactions (see above), so all conformations of each compound were evaluated for the key interactions to identify the candidate docking solution. No energy threshold was defined, meaning that all conformations were evaluated. The lowest-energy conformation of each compound that fulfilled the geometric criteria was stored. Acceptance of a solution required the energy of the hydrogen bond between the protonated amino group in the ligand to D114/3.32 to be −0.2 kcal/mol or lower, that of hydrogen bonds from any ligand atom (typically hydroxyl groups of the catechol) to S193/5.42 and/or S197/5.46 to be −0.2 kcal/mol or lower, and the van der Waals interaction energy between F390/6.52 and aromatic ligand atoms to be −0.3 kcal/mol or lower. These values were generously chosen to allow several docked conformations. For each receptor model, the number of unique agonists that passed the selection scheme was summed up and used to rank the receptor models (Supplementary Table 3). The three top ranked models were further manually assessed based on the geometry of the key interactions, the shape match between the binding pocket and the compounds, and the convergence of structurally similar compounds to a common binding mode. One D_2_R model was selected for further analysis. It had the highest number of accepted docked agonists and ranked third according to receptor score.

### Properties of the selected model

The C_α_ RMSD of the selected D_2_ model compared to the initial (homology) model was 5.4 Å (Supplementary Table 3). Exclusion of TM1 from the analysis, which had clearly different positions in the two models, yielded a C_α_ RMSD of 2.4 Å. Although TM1 shows structural variability across different GPCR structures, the large deviation observed here is probably an artifact caused by the random sampling and the few tethers to this helix. Helix TM1 is not restrained by, nor directly affect, ligand binding properties, and therefore neither the ligand binding score nor the final screening selection will penalize TM1 as long as it retains properties that are membrane protein-like. Superposition of C_α_ atoms of TM2–7 (2.4 Å RMSD) shows that the main structural differences is a sideward shift of the extracellular ends of TM3 and TM4 by 2–3 Å and a corresponding movement in the opposite direction of TM5. This results in a decrease of the distance between the midpoint of the D114/3.32 O_γ_ atoms and S193/5.42 O_γ_ coordinates, from 14 Å in the homology model to 9.1 Å in the selected model, which improves the binding pocket agonist-binding properties (see below). The movements may be triggered by the distance restraints and scoring of receptor-ligand interactions that require helices TM3 and TM5 to move closer, in analogy to the binding of an agonist [16–18]. If the C_α_ atoms of TM3–TM5 are reference points for superposition instead (2.2 Å RMSD) the structural change is a shift of TM2 and TM7 towards TM6 by approximately 3 Å, causing TM6 to tilt out from the bundle center on the intracellular side by 2 Å. The outward shift of TM6 is an important structural feature of the activated state of rhodopsin [19–22], the β_2_AR [17, 18] and the A_2A_ adenosine receptor [15]. The magnitude of the shift in the D_2_R model is modest in comparison to the structures (2 Å vs. 6–11 Å) since the G-protein was not included in the model and also due to the presence of tethers to the inactive state helix packing. Residues I3.40 and F6.44 were suggested to couple conformational changes of the binding pocket with TM6 based on the active agonist-bound β_2_AR structure [17]. These conformational changes are not observed in the D_2_R model, probably due to the inactive-state conformation of the intracellular part of TM6. The binding pocket score of the homology model was inferior relative to that of the selected model. However, the packing score of the homology model was better than those of the models generated by the presented method (see the “Methods/experimental” section for details on the score definitions).

### Binding site analysis

**Fig. 6:**
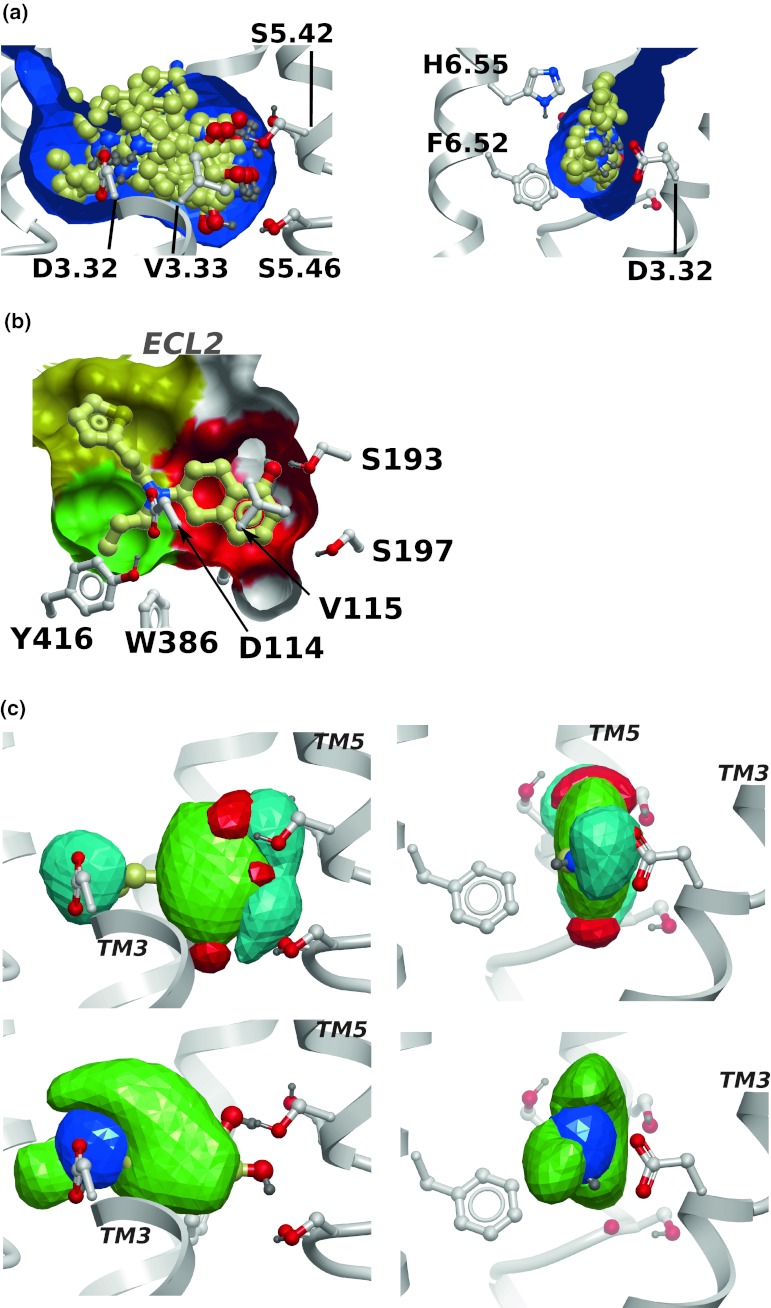
**a** Orthogonal views of ten D_2_R-active compounds docked to the selected D_2_R model showing a good match to the size and shape of binding pocket (*blue surface*). Selected proximal side chains and ribbons were removed for clarity. The pocket shape was calculated using icmPocketFinder [50] which closes the solvent-exposed region, and ligands therefore seems to protrude through the pocket. Selected side chains are labeled. **b** Definition of three regions discussed in the text: The catechol pocket (*red*), the propyl pocket (*green*) and the ECL2-proximal pocket (*yellow*). Other regions of the receptor molecular surface are *white*, selected side chains are shown and labeled, and rotigotine is the representative compound. The amine proton points towards D114/3.32 which is near the viewer. Selected helix numbers are indicated. **c** Orthogonal views of the average atomic property fields (APFs) calculated from docked agonists matching properties of the receptor model. The receptor is shown as ribbon and ball-and-stick models. The surfaces are isocontours for the property fields: sp^2^ hybridized (*green*), hydrogen bond donor (*cyan*) and acceptor (*red*) (*top panels*) and hydrophobic (*green*) and positively charged (*blue*) (*lower panels*). Note the hydrophobic extension near the positive charge that matches the propyl substituents on the basic nitrogen

For further analysis, a more focused set of compounds was used (Fig. 5) by removal of compounds that were structurally similar. Also, compounds that contained large substituents that were expected to interact with the extracellular loop 2 (ECL2) were removed due to the lack of loops in the receptor model. Docking and evaluation using the same criteria as above (geometry of key interactions, shape match and convergence to a common binding mode) resulted in the correct binding mode for all 11 agonists for the selected model. The shape of the binding pocket was calculated using the icmPocketFinder function of ICM that detects both buried and surface-exposed binding sites [50]. This method is useful for the D_2_R model since the ECL2, that closes the pocket in known GPCR structures, is missing. The volume (458 Å^3^) and shape of the binding pocket matches those of the majority of agonists in this study, and the selected docking solutions form a tight cluster (Fig. 6a). The distance from the carboxylate group of D114/3.32 and the O_γ_ hydroxyl of S193/5.42 is 9.1 Å, which is 5.4 Å shorter than that of the unrefined rhodopsin-based homology model. This is in good agreement with the shorter distance between TM3 and TM5 of the activated state that has been suggested [19–22] and later confirmed by structural studies [15, 18]. It also agrees well with the 9.1 Å distance in a pharmacophore model for selective D_2_ agonists, measured from the projected pharmacophoric features representing the serine hydrogen bond donor/acceptor to the aspartic acid projected feature [40]. The corresponding distance in the inverse-agonist bound structure of the dopamine D_3_ receptor is 9.9 Å [13]. Nine out of the ten inactive compounds in the set also matched (all but (−)-DHX), suggesting that the selection criteria and/or the model properties are not sufficient for discrimination between active and inactive compounds in their current form.

### Dopamine D_2_R ligand recognition

Three sub-pockets are present in the binding site of the model (Fig. 6b): (1) A catechol-binding region offers hydrogen bonding interactions to three serine residues in TM5, and aromatic interactions with F390/6.52 in TM6. In addition, V115/3.33 in TM3 is in position to form hydrophobic contacts with the ligand. These receptor-ligand interactions are well established in the literature [45–47, 51, 52]. (2) The model has a small hydrophobic pocket near TM7. Binding studies have shown that a propyl substituent on the basic amino group is important for D_2_R selectivity over the dopamine D_1_ receptor [53–56]. The basic amine can carry two aliphatic substituents, but only one of them can be larger than three carbons or the affinity decreases [54]. An explanation is suggested by the D_2_R model: The hydrophobic pocket near TM7, formed by W386/6.48, T412/7.39, G415/7.42 and Y416/7.43, corresponds in size and shape to an *n*-propyl group. The other substituent projects in the direction of the loops, which are more flexible and may accommodate larger groups. (3) A pocket near the extracellular face of similar size as the catechol-binding pocket. Its size is not well-defined due to the absence of ECL2. However, due to the higher variability of the loop region compared to the helical region, this part is likely to be more flexible than the membrane-buried parts and may adapt to a variety of chemical substituents.

In combination with D114/3.32, these three pockets form a tetrahedral arrangement around the basic amino group of the ligand (Fig. 6b), which allows the binding mode of many catechol-containing monoaminergic ligands to be rationalized. The requirement of a hydrogen bond between the basic amine and D114/3.32 sets a clear directional restraint, which leads to a critical dependence of the stereochemical configuration around the protonated nitrogen for the fit to the binding pocket. Therefore, only one of the *N*-enantiomers was accepted for compounds with a stereogenic protonated nitrogen and a clear difference in size of the *N*-substituents.

In order to generalize the docking results, the properties of the ligand ensemble were represented by so called atom property fields (APFs) [57] for the accepted docked solutions of the agonists. The APFs are 3D grid representations of seven properties that are assigned to each atom: Hydrophobicity, hydrogen bond acceptor, hydrogen bond donor, charge, sp^2^ hybridization, size, and electronegativity. In contrast to the initial APF study, the APFs reported here are based on the docked conformations of the ligands which therefore take receptor interactions into account. As expected, hydrogen bond donor and positive charge fields map to the basic amino group, and aromatic fields are present near TM5, but also near the location of ECL2 (Fig. 6c). The hydrogen bond donor field has an elongated maximum along the ridge of the catechol rings, adjacent to serine residues 193/5.42, 194/5.43 and 197/5.46 on TM5. The hydrogen bond acceptor fields are localized to two lobes on either side of the rings. The presence of propyl substituents on the amines is manifested as an elongated hydrophobic field close to TM7.

As described above, the criterion for the final selection of one out of the nine receptor models is based on the fit of a set of compounds to the ligand binding site. Whereas our aim was that the agonist-biased optimization should favor the active state of the receptor [58], the structural adaptation is not necessarily propagated throughout the whole structure with the current settings. For example, the outward movements of the parts of TM5 and TM6 close to the intracellular side is not of the same magnitude as those of active-state receptors [15, 18]. Although these two states may be partly uncoupled, as shown in the crystal structure of the β_2_AR T4L chimera bound to an inverse agonist while the cytoplasmic end is in the active state [59], tethers to residues in the intracellular region of the receptor may be removed in future work, or set to the corresponding residues of a model in the G-protein interacting state [7].

## Conclusions

During the last few years, progress in 3D structure determination of GPCRs has enabled analysis of ligand recognition by their receptors in different conformational states. The structures of 15 receptors are known at present [14], all belonging to family A, but so far only three agonist-bound active-state structures have been reported (β_2_AR [17, 18] and A_2A_ adenosine receptor [15]). Homology models and ab initio methods have been used to model other receptors, and whereas homology modeling tends to be limited to similar backbone geometries, ab initio methods are generally computationally expensive. Therefore we have combined the two strategies into a semi-empirical method that re-models the helical bundle of a 7TM homology model by Monte Carlo optimization, guided by a ligand and by restraints derived from experimental data, allowing for larger backbone variation while keeping a bias towards the template structure.

A wide conformational space was sampled, as demonstrated by the application of the method to the β_1_AR and D_2_R receptors. The automatic docking of agonists to the selected D_2_R receptor model converged to a common binding mode for several compounds, and alternative binding modes were selected manually from the docking conformational stacks to yield a well-defined model for agonist binding. The structural features of docked ligands were interpreted using APF, and they correlated well with experimental data. The binding site has been biased towards the agonist-bound state by the use of constraints based on receptor-agonist key interactions. However, these conformational changes were not propagated to the inactive state R132/3.50–E368/6.30 ionic lock on the intracellular side, probably due to the constraints to conserved residues of the homology model. Therefore, the analysis is restricted to the ligand-binding site in the current form of the method. It is however possible to bias the structure optimization to several structure templates depending on the desired properties—e.g. using the binding pockets of monoaminergic receptors or the intracellular part of G-protein peptide-bound structure models [7, 18].

This type of semi-empirical approach has a potential for modeling of receptor targets for which a structural template with sufficient sequence homology or the correct conformational state is unavailable, but for which sufficient empirical information is accessible to guide the modeling.

## Methods/experimental

### General

Default ICM energy terms and parameters were used for energy evaluations [60]. The maximal van der Waals interaction energy in the first stage of structure optimization was limited to 2.0 kcal/mol. A distance-dependent dielectric constant of 4 was used. Template structures were converted to ideal covalent geometry prior to homology model building to conform with the ICM internal coordinate force field [60]. Homology models were built using standard techniques implemented in ICM.

Since the focus of this study was ligand recognition, the torsion variables of the ligand binding region were sampled more often in the Monte Carlo protocol. Variables of the ligand (torsions and positional variables) were sampled twice as often as those of the binding pocket (defined as residues within 7 Å from the ligand in this context), five times more often than helix positional variables and 50 times more often than the remaining protein residues. The binding pocket residues were re-defined regularly during the optimization to account for structural changes.

All calculations were performed on a dual Intel Xeon workstation running Linux. The generation of ten final models (stage 4) from a single homology model typically required 4–5 days on this single computer, and the docking of ligands and automatic evaluation required 1 day. Covering the corresponding conformational space using molecular dynamics simulations would require significantly larger computational resources.

### Protein/ligand complex scoring

The qualities of the generated complex models need to be assessed during and after the docking part. We therefore developed a series of functions that score the protein and ligand geometries and energies. Protein-related scoring terms are aimed to evaluate helical membrane protein structures in general and are related to a) the total protein molecular surface area and volume, the distance between helix centra, the number and volume of internal cavities, and b) the tilt of helices relative to the overall bundle orientation, the offset of helix centra from the mid-plane of the bundle (parallel to the membrane), the distances between helix ends relative to loop lengths, and the distance between the polar residues that anchor distal parts of the ligand. The weighted sum of terms in a) is referred to as the protein packing score, whereas that of b) is the protein orientation score. The ligand score is target-specific and depends on the chemical properties of the compound and its interactions with the receptor. The three scores are weighted and summed into a total score for the complex. The development of each term and determination of weight factors were based on their application to a selected set of determined membrane protein structures and decoy 7TM models where helical orientations were partially randomized. Since it was not known a priori which functions would give meaningful measures of quality, we allowed overlapping functions be included.

#### Packing

The terms that correlate with packing include the following: *Total molecular volume and area*—tightly packed complexes tend to have smaller volumes and areas. *Number and volume of pockets*—badly packed cores contain buried pockets that were predicted by ICM’s pocket finder. Pockets located on the surface of the receptor are also predicted by the method but are not related to packing defects, which leads to noise in this term. *Helix center distance*—The distances between the geometric centers of each helix and the bundle centre are summed. An offset of a helix along its helical axis relative to the bundle will increase this term, and it is generally a good measure of packing if residue ranges are properly chosen.

#### Orientation

*Hydrophilic surface* Hydrophilic residue side chains are in general in contact with other protein residues and hidden from the lipid surrounding. The exposed area of hydrophilic residues was calculated for residues in helix conformation considering the solvent exposed area of (1) those charged and polar groups that were at least 25 % surface-exposed relative to their standard exposed area in a Gly-X-Gly tripeptide [60] and (2) all charged and polar residues including also those of small surface-exposed area. Only the most membrane-embedded mid-third section of the bundle was considered. *Tilt* Strongly tilted helices are rare in membrane proteins, with the exception of shorter segments near the membrane surface. Helix segments that were tilted more than 50 ° compared to the bundle axis were penalized by an amount proportional to the tilt. Helices are assigned prior to this calculation, based on the ICM assignment (a modification of the DSSP [61] algorithm). This is followed by a more conservative re-evaluation by breaking helices into segments at helical kinks based on the distance between carbonyl oxygens in the backbone. The overall orientation vector of the bundle was determined by the sum of the individual helix vectors, weighted by the number of residues in each helix. All helical vectors were clustered prior to the overall bundle axis determination, and up to 10 % of the helices that were not in the main cluster were neglected to avoid the contribution from helices that deviated from the main bundle vector. *Elevation* Elevation is the calculated distance offset of helix centers from the membrane mid-plane and should be near zero if helix regions are accurately modeled. *Loops* The distance between helix termini is not explicitly limited during the docking since loops are not included in models. Preliminary attempts to use distance restraints as replacements for loops were not satisfactory, instead we introduced a scoring term based on the difference between the distance between the helical end residues and the expected maximal length of the loop (estimated using 3.2 Å per residue). *Distance between ligand anchor residues* A flat-bottom quadratic function was used to score the distance between key interacting residues that anchor distal parts of the ligands. The target distance was estimated from ligand geometry, as described for each case above.

#### Ligand

The ligand score is calculated from receptor-ligand interaction energies and is defined similarly for each complex, see below.

### Ligand docking to sets of receptor models

For the ligand docking to sets of receptor models, docking parameters were set up for one protein–ligand complex model and used for all other models, except for the exact selection of receptor atoms. The atom selection was made using a 10 × 10 × 10 Å^3^ box with defined coordinates for one model, and other receptor models were superimposed with the ICM “align” sequence/structure alignment tool which aligns those parts of the 3D model that are conserved in sequence. This means that single helices that differ in orientation relative to the folded core will not affect the structural alignment and binding site selection.

### Turkey β_1_AR test case

The structure prediction method was developed using the turkey β_1_AR as target, and the bovine rhodopsin crystal structure (pdb entry 1u19) [5] as the template. The helical sequence regions W40-S68, L75-V103, G110-A142, R155-I177, R205-E233, H286, V314, D322-Y343 were used to build a homology model using ICM. The inverse agonist (*S*)-cyanopindolol was generated from 2D coordinates using ICM and added to the receptor model. In the scoring evaluation, the non-penalized distance from the average coordinate of the O_δ_ atoms of D121/3.32 to the average coordinate of the O_γ_ atoms of S211/5.42 and S215/5.46 was set to 9.5 ± 1.5 Å. The corresponding distance is 10.8 Å in the crystal structure. The ligand score was the weighted sum of the following terms: Hydrogen bond energy between the ligand and serine residues S211/5.42 and S215/5.46; electrostatic and hydrogen bond energies between the ligand and the side chain atoms of D121/3.32; distance restraint energies from two heavy atoms of the ligand and three receptor atoms (Fig. 3a), and van der Waals interactions between ligand aromatic atoms and F06/6.51,F307/6.52 that substitutes for aromatic face-to-edge π–π interactions (see “Results and discussion”). Too large conformational variation was observed for TM7 which was due to the lack of restraints at its N-terminal half. Only four residues at the extreme C-terminus are conserved in the 22 residue helix. Therefore one extra restraint from the model N-terminus to the template was defined for the N329/7.39 C_α_ atom, yielding a total number of restraints of 5, 7, 9, 3, 7, 7 and 5 for TM1-TM7, respectively. The 19 residues that were within 5 Å from the ligand in the crystal structure were defined as binding site residues. Receptor and ligand RMSDs for each model were calculated after superposition of the binding site residues.

### Dopamine D_2_ modeling

The modeled helical regions were Y34-E62, T67-V97, F102-T134, K149-G173, A185-K211, E368-D400, P405-I425. In the scoring evaluation, the non-penalized distance from the average coordinate of the O_δ_ atoms of D114/3.32 to the average coordinate of the O_γ_ atoms of S193/5.42 and S197/5.46 was set to 12 ± 2 Å estimated from the corresponding distance (15 Å) in the homology model and considering the longest distance between the catechol oxygens and the nitrogen of (*R*)-NPA (8 Å). The ligand score was the weighted sum of the following terms: Hydrogen bond energy between the ligand and serine residues S193/5.42 and S197/5.46; electrostatic and hydrogen bond energies between the ligand and the side chain atoms of D114/3.32; distance restraint energies from two heavy atoms of the ligand and three receptor atoms (Fig. 3b), and van der Waals interactions between ligand aromatic atoms and F389/6.51,F390/6.52 that substitutes for aromatic face-to-edge π-π interactions (see “Results and discussion”). The distribution of C_α_ RMSD from all models to the template was 3.5–5 Å for about 90 % of the solutions, and above 5 Å for the remaining fraction.

## Electronic supplementary material

Below is the link to the electronic supplementary material.

Supplementary material 1 (PDF 424 kb)

## Abbreviations

3D: Three dimensional
7TM: Seven transmembrane helix
APF: Atomic property fields
β_1_AR: β_1_ Adrenergic receptor
β_2_AR: β_2_ Adrenergic receptor
D_2_R: Dopamine D_2_ receptor
ECL2: Extracellular loop 2
GPCR: G-protein coupled receptor
PDB: Protein data bank
RMSD: Root mean square deviation
TM: Transmembrane
TM1, …, TM7: Transmembrane helix 1, …, 7
VLS: Virtual ligand screening
