# Supplementary material for: Development of 7TM receptor-ligand complex models using ligand-biased, semi-empirical helix-bundle repacking in torsion space: application to the agonist interaction of the human dopamine D2 receptor
Source: J Comput Aided Mol Des. 2013 Apr 4;27(3):277–91. doi: 10.1007/s10822-013-9640-z (PMC3639355; doi:10.1007/s10822-013-9640-z)
Supplement: Supplementary file 1 — Supplementary material 1 (PDF 424 kb) [file 10822_2013_9640_MOESM1_ESM.pdf]

Supplementary material

Development of 7TM receptor-ligand complex models using ligand-biased, semi-empirical helix-bundle repacking in torsion space: Application to the agonist interaction of the human dopamine D_2 receptor

Marcus Malo[1†], Ronnie Persson[1†], Peder Svensson[2], Kristina Luthman[1], Lars Brive[1,3,4*]

[1] Dept. of Chemistry and Molecular Biology, University of Gothenburg, SE-412 96 Göteborg, Sweden

[2] NeuroSearch Sweden AB, Arvid Wallgrens Backe 20, SE-413 46 Göteborg, Sweden

[3] Dept of Medical Biochemistry, University of Gothenburg, Box 440, SE- 405 30 Göteborg, Sweden

[4] Current address: Cygnal Bioscience, Björnvägen 15, SE-435 43 Pixbo, Sweden

[†] These authors contributed equally to this work

Running title: 7TM receptor-ligand structure repacking

***Corresponding author:**

Lars Brive

Cygnal Bioscience, Björnvägen 15, SE-435 43 Pixbo, Sweden

Phone: +46-31-3364466; Fax +46-31-3360639

Email: lars.brive@cygnal.se

Supplementary Table 1.

RMSD and score values for final 10 β_1AR receptor models derived from the bovine rhodopsin structure, versus the crystal structure (PDB id 2vt4 [1]). (*S*)-Cyanopindolol was docked three times to each receptor model. RMSDs were calculated for all C_α carbons, or for heavy atoms of binding site residues within a 4 Å radius from the ligand of the crystal structure. The receptor binding site superposition was used for calculation of ligand heavy atom RMSDs. The methyl carbon atoms of the *t*-butyl group of the ligand were excluded from the RMSD calculation to avoid their incorrect pairwise matching.

	Score		RMSD	
Complex	site	Cα	site	ligand
10.31	9.35	2.8	1.6	1.6
				1.6
				1.5
10.22	9.46	5.5	1.6	0.5
				0.5
				0.5
10.03	9.40	3.3	2.3	5.9
				5.9
				5.9
10.02	9.49	4.0	2.1	4.6
				4.6
				4.6
9.97	9.95	3.0	2.6	3.4
				3.3
				3.3
9.90	9.17	2.4	1.8	1.4
				1.4
				1.4
9.88	8.68	2.8	2.0	1.7
				1.7
				1.7
9.85	8.98	3.6	2.8	5.3
				5.3
				5.2
9.78	10.29	5.4	1.6	1.6
				1.6
				1.6
9.16	10.10	4.1	2.1	5.5
				5.5
				5.5
homology model	-6.0	2.9	1.7	7.1
				6.5
				3.0

Supplementary Table 2.
Compounds used for docking and selection of receptor model. A selection of these compounds were docked to the final model and analyzed in more detail (Fig. 5).

Name	Reference
Active compounds	
U-86170	[2]
(*R*)-Sumanirole	[3]
Rotigotine	[4]
PTAT	[5]
nPr-DHX	[6]
(*R*)-NPA	[7]
(*R*,*R*)-PHNO	[8]
(3a*S*,9a*S*)-1-Propyl-2,3,3a,4,9,9a-hexahydro-1*H*-benzo[*f*]indole-5-ol ((-)-**1**)	[9]
(3a*S*,9a*S*)-1-(2-Propenyl)-2,3,3a,4,9,9a-hexahydro-l*H*-benz[*f*lindole-5-ol ((-)-**2**)	[9]
(*S*)-PPHT	[10]
(*S*)-5-OH-DPAT	[5]
Apomorphine	[7]
Dopamine	
Talipexole	[11]
Quinpirole	[12]
Pramipexole	[13]
U-91356A	[14]
(*S*)-DPAT	[15]
(*R*)-(+)-3-PPP[a]	[16]
A-70108	[17]
Inactive compounds	
A-70360	[18]
(*S*,*S*)-PHNO	[8]
(3a*R*,9a*R*)-1-Propyl-2,3,3a,4,9,9a-hexahydro-1*H*-benzo[*f*]indol-5-ol ((+)-**1**)	[9]
cis-DHX	[6]
(-)-DHX	[19]
(*S*)-7-OH-DPAT	[15]
SKF38393	[20] and references therein
Doxanthrine[a]	[21]
trans-10-Bromo-11-hydroxy-6-methyl-5,6,6a,7,8,12b-hexahydrobenzo[*a*]phenanthridine (**3**)	[22]
(*R*)-5-OH-DPAT	[23]
A77636	[24]
(*S*)-Sumanirole[a]	[25]

[a] Only used in the analysis of the final receptor model.

Supplementary Table 3.

Docking of 29 compounds to D_2R structure models. A total of 43 compounds were docked if both *N*-enantiomeric forms are considered. The number of fully active or inactive compounds that had a solution that was considered a hit by the automatic scoring function is shown. The model with the highest number of matching agonists was selected after manual inspection of the top three solutions. No successful docking solutions were identified for the unrefined homology model which was the source of all other models. This was primarily due to a rotation of the D114 side-chain being rotated away from the binding pocket which restricts salt bridge formation with the ligand. The RMSD is calculated for C_α of TM helices and for binding pocket heavy atoms, relative to the initial D_2R homology model.

Complex score	active (max 19)	inactive (max 10)	RMSD (C_α) TM 3,5,6,7/TM 1-7	RMSD (non-H) binding pocket
-4.41	11	8	2.5/5.7	1.9
-4.36[a]	14	9	2.2/5.4	2.4
-4.36	13	9	2.4/6.2	2.2
-3.81	7	6	2.9/5.1	2.4
-4.80	5	4	3.2/3.7	3.4
-4.32	8	2	2.2/3.8	2.8
-4.28	4	5	2.6/3.2	2.7
-4.20	1	0	3.0/5.0	2.5
-3.25	0	0	2.1/3.7	1.7
-1.44[b]	0	0	0.0/0.0	0.0

[a] Selected model; [b] Homology model

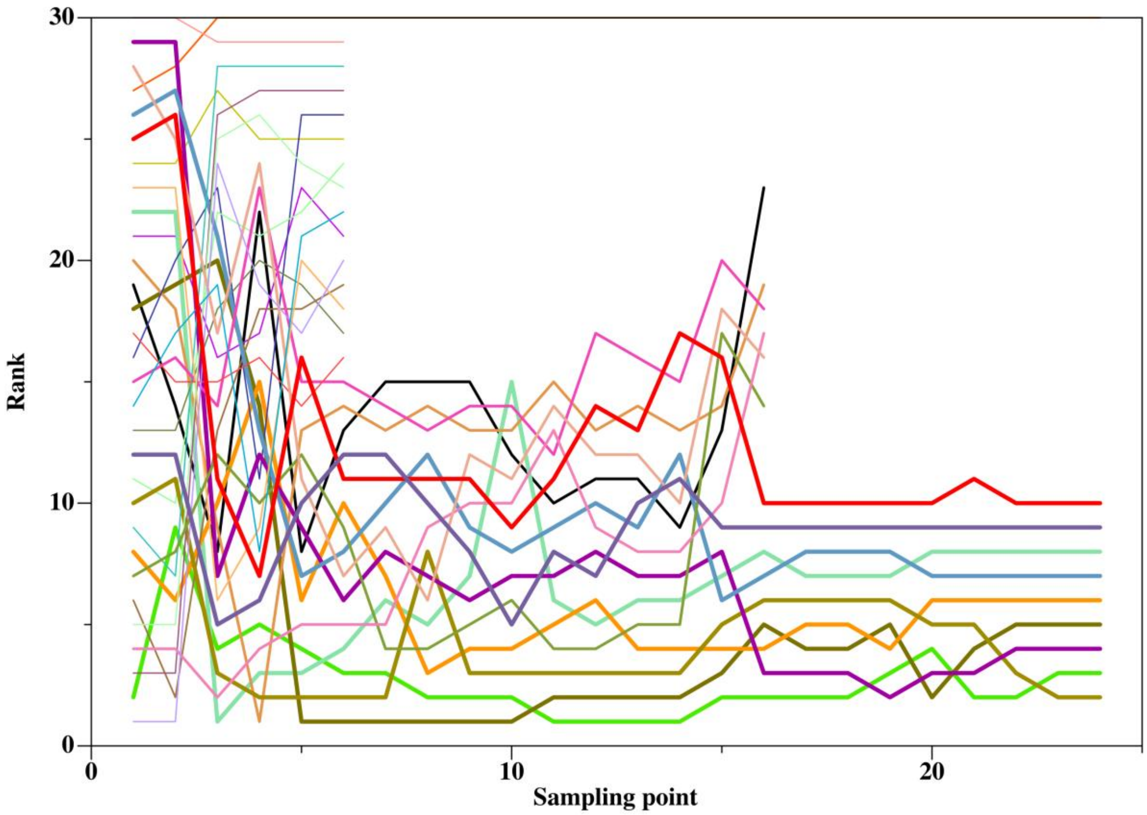

Supplementary Figure 1. Plot of the rank of each model relative to all others versus the Monte Carlo optimization progress, which shows that the best ranked models in the early phase in general rank among the best also at the end of the optimization. The removal of models with bad score in the early phases (at sampling points 6 and 16 in this case) improved computational performance. Although good solutions were rarely lost using this strategy, in this case the best ranked final solution was removed by the first filter. All 30 models were run until completion in this experiment, but the rank is only shown for those that would qualify at the two thresholds.

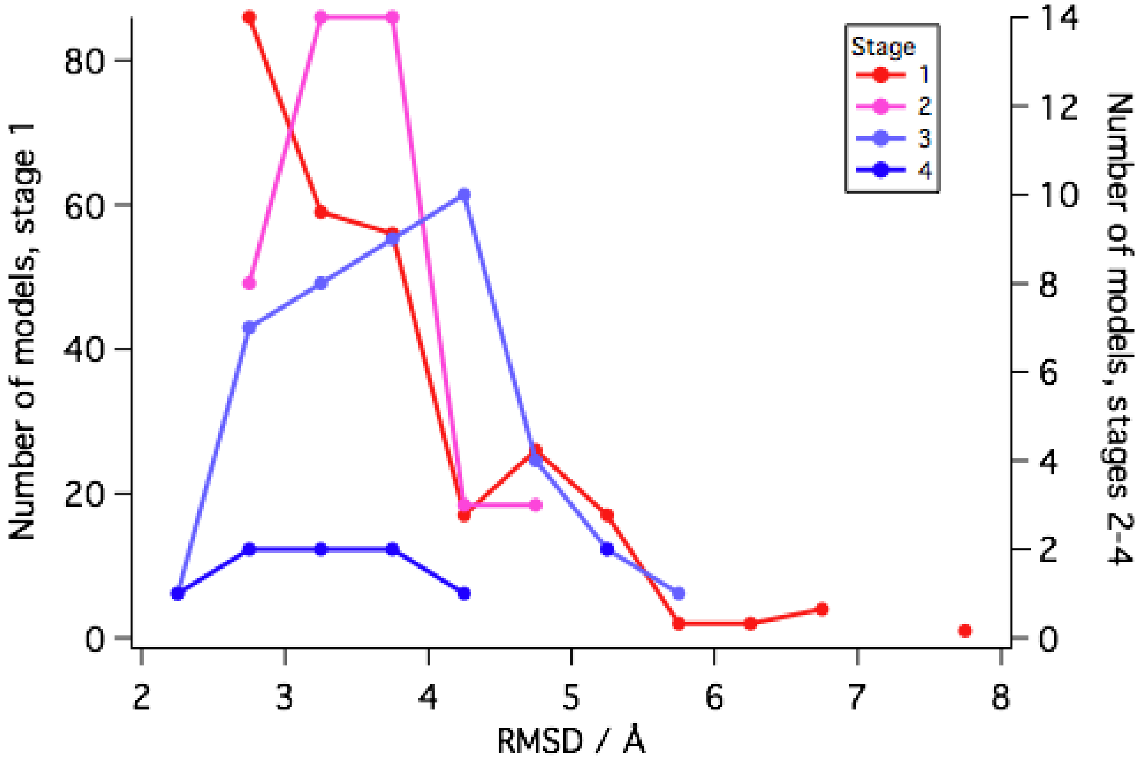

Supplementary Figure 2. Distribution of C_α RMSD values between β_1 adrenergic receptor models generated from the rhodopsin-based homology model and the crystal structure of β_1 adrenergic receptor, both in complex with (*S*)-cyanopindolol. Stages 1-4 are shown individually with color coding according to the legend, and each marker represents the number of models binned in RMSD intervals with 0.5 Å width. Only values above 0 are shown, resulting in discontinued lines for stage 1 and 4. Other measures of model quality, e.g. ligand docking geometry, are shown elsewhere. The total number of models was 364.

```
1u19_a     1    MNGTEGPNFYVPFSNKTGVVRSPFEAPQYYLAEPWQFSMLAAYMFLLIMLGFPINFLTLYV
DRD2       1    MDPLNLSWYDDDLERQNWSRPFNGSDGKADRPHYNYYATLLTLLIAVIVFGNVLVCMAVSR
D2model         ---------------------------------YNYYATLLTLLIAVIVFGNVLVCMAVSR
1u19_a

1u19_a     62   TVQHKKLRTPLNYILLNLAVADLFMVFGGFTTTLYTSLHGYFVFGPTGCNLEGFFATLGGE
DRD2       62   E---KALQTTTNYLIVSLAVADLLVATLVMPWVVYLEVVGEWKFSRIHCDIFVTLDVMMCT
D2model         E-------TTTNYLIVSLAVADLLVATLVMPWVVYLEVV----FSRIHCDIFVTLDVMMCT
1u19_a

1u19_a     123  IALWSLVVLAIERYVVVCKPMS-NFRFG-ENHAIMGVAFTWVMALACAAPPLVGWSRYIPE
DRD2       120  ASILNLCAISIDRYTAVAMPMLYNTRYSSKRRVTVMISIVWVLSFTISCPLLFGLNN--AD
D2model         ASILNLCAISIDRYT--------------KRRVTVMISIVWVLSFTISCPLLFG--------
1u19_a

1u19_a     182  GMQCSCGIDYYTPHEETNNESFVIYMFVVHFIIPLIVIFFCYGQLVFTVKEAAAQ------
DRD2       179  QNECII-----------ANPAFVVYSSIVSFYVPFIVTLLVYIKIYIVLRRRRKRVNTKRS
D2model         -----------------ANPAFVVYSSIVSFYVPFIVTLLVYIK-----------------
1u19_a

1u19_a     236  -------------------------------------------------------------
DRD2       229  SRAFRAHLRAPLKGNCTHPEDMKLCTVIMKSNGSFPVNRRRVEAARRAQELEMEMLSSTSP
D2model         -------------------------------------------------------------
1u19_a

1u19_a     236  -------------------------------------------------------------
DRD2       290  PERTRYSPIPPSHHQLTLPDPSHHGLHSTPDSPAKPEKNGHAKDHPKIAKIFEIQTMPNGK
D2model         -------------------------------------------------------------
1u19_a

1u19_a     236  -------QQESATTQKAEKEVTRMVIIMVIAFLICWLPYAGVAFYIFTHQGSDFGPIFMTI
DRD2       351  TRTSLKTMSRRKLSQQKEKKATQMLAIVLGVFIICWLPFFITHILNIHCD-CNIPPVLYSA
D2model         -----------------EKKATQMLAIVLGVFIICWLPFFITHILNIHCD-----PVLYSA
1u19_a

1u19_a     291  PAFFAKTSAVYNPVIYIMMNKQFRNCMVTTLCCGKNPLGDDEASTTVSKTETSQVAPA
DRD2       411  FTWLGYVNSAVNPIIYTTFNIEFRKAFLKILHC-------------------------
D2model         FTWLGYVNSAVNPII-------------------------------------------
1u19_a
```

Supplementary Figure 3. Multiple sequence alignment of the homology modeling template bovine rhodopsin chain A (1u19_a; PDB entry 1u19 [26]), the human D_2R long (DRD2; SWISS-PROT entry DRD2_HUMAN) and the modeled D_2R TM regions (D2model). The conservation strength is color coded from white through green, and the secondary structure of rhodopsin, as described in the pdb entry, is indicated by red (α-helix), magenta (3/10 helix) and green (β strand) bars and arrows. The *C*-terminal helix TM8 is parallel to the membrane plane and was not modeled. Manual adjustment of the alignment was only made for the regions that were modeled.

References

1. Warne T, Serrano-Vega MJ, Baker JG, Moukhametzianov R, Edwards PC, Henderson R, Leslie AG, Tate CG, Schertler GF (2008) Structure of a beta1-adrenergic G-protein-coupled receptor. Nature 454:486-491

2. Moon MW, Morris JK, Heier RF, Chidester CG, Hoffmann WE, Piercey MF, Althaus JS, Von Voigtlander PF, Evans DL, Figur LM, Lahtis RA (1992) Dopaminergic and serotonergic activities of imidazoquinolinones and related compounds. J Med Chem 35:1076-1092

3. Lahti RA, Figur LM, Piercey MF, Ruppel PL, Evans DL (1992) Intrinsic Activity Determinations at the Dopamine D2 Guanine Nucleotide-Binding Protein-Coupled Receptor - Utilization of Receptor State Binding Affinities. Mol Pharmacol 42:432-438

4. Copinga S, Dijkstra D, Devries JB, Grol CJ, Horn AS (1993) Synthesis and Pharmacological Evaluation of 5,6,7,8-Tetrahydro-6-[Propyl[2-(2-Thienyl)Ethyl]Amino]-1,2-Naphthalenedi Ol - a Novel Nonselective Dopamine-Receptor Agonist. J Med Chem 112:137-142

5. Venhuis BJ, Dijkstra D, Wustrow DJ, Meltzer LT, Wise LD, Johnson SJ, Heffner TG, Wikstrom HV (2003) Orally active analogues of the dopaminergic prodrug 6-(N,N-di-n-propylamino)-3,4,5,6,7,8-hexahydro-2H-naphthalen-1-one: Synthesis and pharmacological activity. J Med Chem 46:584-590

6. Mottola DM, Kilts JD, Lewis MM, Connery HS, Walker QD, Jones SR, Booth RG, Hyslop DK, Piercey M, Wightman RM, Lawler CP, Nichols DE, Mailman RB (2002) Functional selectivity of dopamine receptor agonists. I. Selective activation of postsynaptic dopamine D-2 receptors linked to adenylate cyclase. J Pharm Exp Ther 301:1166-1178

7. Goldman ME, Kebabian JW (1984) Aporphine Enantiomers - Interactions with D-1 and D-2 Dopamine-Receptors. Mol Pharmacol 25:18-23

8. Seeman P, Ulpian C, Larsen RD, Anderson PS (1993) Dopamine-Receptors Labeled by PHNO. Synapse 14:254-262

9. Lin CH, Haadsma-Svensson SR, Phillips G, Lahti RA, McCall RB, Piercey MF, Schreur PJ, Von Voigtlander PF, Smith MW, Chidester CG (1993) Centrally acting serotonergic and dopaminergic agents. 2. Synthesis and structure-activity relationships of 2,3,3a,4,9,9a-hexahydro-1H-benz[f]indole derivatives. J Med Chem 36:1069-1083

10. Bakthavachalam V, Baindur N, Madras BK, Neumeyer JL (1991) Fluorescent probes for dopamine receptors: synthesis and characterization of fluorescein and 7-nitrobenz-2-oxa-1,3-diazol-4-yl conjugates of D-1 and D-2 receptor ligands. J Med Chem 34:3235-3241

11. Mewshaw RE, Kavanagh J, Stack G, Marquis KL, Shi XJ, Kagan MZ, Webb MB, Katz AH, Park A, Kang YH, Abou-Gharbia M, Scerni R, Wasik T, Cortes-Burgos L, Spangler T, Brennan JA, Piesla M, Mazandarani H, Cockett MI, Ochalski R, Coupet J, Andree TH (1997) New generation dopaminergic agents .1. Discovery of a novel scaffold which embraces the D-2 agonist pharmacophore. Structure-activity relationships of a series of 2-(aminomethyl)chromans. J Med Chem 40:4235-4256

12. Seeman P, Schaus JM (1991) Dopamine-Receptors Labeled by [3H]Quinpirole. Eur J Pharmacol 203:105-109

13. Sethy VH, Ellerbrock BR, Wu H (1997) U-95666E: a potential anti-parkinsonian drug with anxiolytic activity. Prog Neuropsychopharmacol Biol Psychiatry 21:873-883

14. Piercey MF, Moon MW, Sethy VH, Schreur PJ, Smith MW, Tang AH, Von Voigtlander PF (1996) Pharmacology of U-91356A, an agonist for the dopamine D2 receptor subtype. Eur J Pharmacol 317:29-38

15. Payne SL, Johansson AM, Strange PG (2002) Mechanisms of ligand binding and efficacy at the human D-2(short) dopamine receptor. J Neurochem 82:1106-1117

16. Hjorth S, Carlsson A, Clark D, Svensson K, Wikstrom H, Sanchez D, Lindberg P, Hacksell U, Arvidsson LE, Johansson A, et al. (1983) Central dopamine receptor agonist and antagonist actions of the enantiomers of 3-PPP. Psychopharmacology (Berl) 81:89-99

17. DeNinno MP, Schoenleber R, Perner RJ, Lijewski L, Asin KE, Britton DR, MacKenzie R, Kebabian JW (1991) Synthesis and dopaminergic activity of 3-substituted 1-(aminomethyl)-3,4-dihydro-5,6-dihydroxy-1H-2-benzopyrans: characterization of an auxiliary binding region in the D1 receptor. J Med Chem 34:2561-2569

18. DeNinno MP, Schoenleber R, Asin KE, MacKenzie R, Kebabian JW (1990) (1R,3S)-1-(Aminomethyl)-3,4-dihydro-5,6-dihydroxy-3-phenyl-1H-2-benzopyran: a potent and selective D1 agonist. J Med Chem 33:2948-2950

19. Knoerzer TA, Nichols DE, Brewster WK, Watts VJ, Mottola D, Mailman RB (1994) Dopaminergic Benzo[*a*]phenanthridines - Resolution and Pharmacological Evaluation of the Enantiomers of Dihydrexidine, the Full Efficacy D-1 Dopamine-Receptor Agonist. J Med Chem 37:2453-2460

20. Oboyle KM, Waddington JL (1984) Selective and Stereospecific Interactions of R-SK&F 38393 with [H-3]-Labeled Piflutixol but Not [3H]Spiperone Binding to Striatal D1-Dopamine and D2-Dopamine Receptors - Comparisons with SCH-23390. Eur J Pharmacol 98:433-436

21. Cueva JP, Giorgioni G, Grubbs RA, Chemel BR, Watts VJ, Nichols DE (2006) trans-2,3-Dihydroxy-6a,7,8,12b-tetrahydro-6H-chromeno[3,4-*c*]isoquinoline: Synthesis, resolution, and preliminary pharmacological characterization of a new dopamine D-1 receptor full agonist. J Med Chem 49:6848-6857

22. Brewster WK, Nichols DE, Watts VJ, Riggs RM, Mottola D, Mailman RB (1995) Evaluation of Cis and Trans-9 and 11-Hydroxy-5,6,6a,7,8,12b-hexahydrobenzo[*a*]phenanthridines as Structurally Rigid, Selective D-1 Dopamine-Receptor Ligands. J Med Chem 38:318-327

23. Malmberg A, Mohell N, Backlund Hook B, Johansson AM, Hacksell U, Nordvall G (1998) Interactions of ligands with active and inactive conformations of the dopamine D2 receptor. Eur J Pharmacol 346:299-307

24. Ryman-Rasmussen JP, Nichols DE, Mailman RB (2005) Differential activation of adenylate cyclase and receptor internalization by novel dopamine D1 receptor agonists. Mol Pharmacol 68:1039-1048

25. Heier RF, Dolak LA, Duncan JN, Hyslop DK, Lipton MF, Martin IJ, Mauragis MA, Piercey MF, Nichols NF, Schreur PJ, Smith MW, Moon MW (1997) Synthesis and biological activities of (R)-5,6-dihydro-N,N-dimethyl-4H-imidazo[4,5,1-ij]quinolin-5-amine and its metabolites. J Med Chem 40:639-646

26. Okada T, Sugihara M, Bondar AN, Elstner M, Entel P, Buss V (2004) The retinal conformation and its environment in rhodopsin in light of a new 2.2 A crystal structure. J Mol Biol 342:571-583
